# Ants from the tropical dry forest in the Cauca River geographic valley, Colombia: new records and expansion of distributions

**DOI:** 10.3897/BDJ.13.e151722

**Published:** 2025-05-19

**Authors:** Maria Alejandra Bautista-Giraldo, Emira I. García, Inge Armbrecht, Roberto J. Guerrero

**Affiliations:** 1 Universidad del Valle, Santiago de Cali, Colombia Universidad del Valle Santiago de Cali Colombia; 2 Universidad del Magdalena, Santa Marta, Colombia Universidad del Magdalena Santa Marta Colombia

**Keywords:** biodiversity, exotic species, Formicidae, habitat fragmentation, native species, secondary forests

## Abstract

**Background:**

Tropical dry forests (TDF) are amongst the most important tropical biomes globally, recognised for their remarkable biodiversity. This biome features a favourable climate and high soil fertility, which has facilitated the development and expansion of human societies. However, continuous and extensive human intervention has led to habitat loss and fragmentation, disrupting the assemblages of biological communities. Within these communities, ants (Hymenoptera, Formicidae) represent one of the predominant groups and their high sensitivity to habitat transformation makes them a useful model for monitoring the effects of land-use changes and assessing ecosystem quality. The objective of this research was to compare ant diversity between two habitat types, tropical dry forest fragments and the surrounding matrices in the Cauca River Valley region. Ants were collected from 2021 to 2022 using six collection methods: arboreal baiting, epigeal baiting, hypogeal baiting, pitfall traps, mini-Winkler extractors and manual capture. A substantial portion of the data generated from this research is available in two datasets associated with two Colombian entomological collections. The first dataset (1574 ant records) is housed at the Centro de Colecciones Biológicas de la Universidad de Magdalena (CBUMAG) and the second (546 records) at the Museo de Entomología de la Universidad del Valle (MUSENUV).

**New information:**

Five ant species were registered for the first time in Colombia: *Leptogenysmontuosa* Lattke, 2011, *Neoponerarugosula* Emery, 1902, *Neoponerazuparkoi* Mackay & Mackay, 2010, *Pheidoletraini* Wilson, 2003 and *Wasmanniasulcaticeps* Emery, 1894. Additionally, the distributions of 45 ant species native to two Departments in the Cauca River Valley geographic region (VGRC) have been expanded. Of these, 31 species extended their distribution into the Valle del Cauca Department: *Aztecaalfari* Emery, 1893, *Foreliusdamiani* Guerrero & Fernández, 2008, *Ecitonmexicanum* Roger, 1863, *Neivamyrmexemersoni* (Wheeler, 1921), *Holcoponeramoelleri* Forel, 1912, *Brachymyrmexmusculus* Forel, 1899, *Cephalotesporrasi* (Wheeler, 1942), *Cephalotestargionii* (Emery, 1894), *Cephalotesumbraculatus* (Fabricius, 1804), *Crematogastermontezumia* Smith, 1858, *Crematogasterobscurata* Emery, 1895, *Nesomyrmexpittieri* (Forel, 1899), *Octostrumabatesi* (Emery, 1894), *Octostrumaexcertirugis* Longino, 2013, *Octostrumaobtusidens* Longino, 2013, *Pheidolebilimeki* Mayr, 1870, *Pheidoleboliviana* Wilson, 2003, *Pheidolecolobopsis* Mann, 1916, *Pheidolegauthieri* Forel, 1901, *Pheidolehasticeps* Wilson, 2003, *Pheidolesimonsi* Wilson, 2003, *Pheidolesubarmata* Mayr, 1884, *Pheidolevallifica* Forel, 1901, *Solenopsisazteca* Forel, 1893, *Solenopsisbrevicornis* Emery, 1888, *Strumigenysgrytava* (Bolton, 2000), *Strumigenysmarginiventris* Santschi, 1931, *Strumigenyswheeleriana* Baroni Urbani, 2007, *Temnothoraxsubditivus* (Wheeler, 1903), *Hypoponeraopacior* (Forel, 1893), *Leptogenyspubiceps* Emery, 1890 and *Pseudomyrmexlongior* (Forel, 1904). Ten species extended their distribution into the Cauca Department: *Aztecavelox* Forel, 1899, *Brachymyrmexminutus* Forel, 1893, *Crematogasterevallans* Forel, 1907, *Megalomyrmexdrifti* Kempf, 1961, *Pheidolefimbriata* Roger, 1863, *Pheidolelongiscapa* Forel, 1901, *Pheidoleradoszkowskii* Mayr, 1884 and *Rogeriascandens* (Mann, 1922); and four species extended their distribution into both Departments: *Brachymyrmexcordemoyi* Forel, 1895, *Pheidolesculptior* Forel, 1893, *Hypoponerafiebrigiantoniensis* (Forel, 1912) and *Hypoponeraparva* (Forel, 1909). Finally, three exotic species were registered for the first time in the VGRC, extending their distributions within Colombia: *Strumigenysemmae* (Emery, 1890) in the Valle del Cauca Department and *Cardiocondylaemeryi* Forel, 1881 and *Cardiocondylawroughtonii* (Forel, 1890) in the Cauca Department.

## Introduction

The Tropical Dry Forest (TDF) is a key ecosystem, characterised by a distinctive flora and fauna, which results in a unique and differentiated biodiversity (Dirzo R. et al. 2011, Portillo-Quintero et al. 2015, Hernández-Jaramillo et al. 2018). However, it is a seriously threatened ecosystem, with less than 10% of its original extension remaining in many countries in both Latin America and the Caribbean (Dryflor et al. 2016), therefore placing it as one of the most threatened ecosystems worldwide (Janzen 1988).

In Colombia, the situation is even more critical. It is estimated that the TDF originally covered over nine million hectares. Still, today, only about up to 8% of its original extent remains (Arcila Cardona et al. 2012), due to agricultural expansion, as well as the growth of human settlements in the lowlands where TDF is distributed (Pizano and García 2014). Habitat loss and degradation are attributed to continuous human intervention, driven by activities, such as agriculture, livestock farming, deforestation and the establishment and expansion of urban settlements (García Guzmán et al. 2016).

The distribution of the TDF in Colombia is not homogeneous. Currently, there are six major regions distributed throughout Colombian territory (Pizano and García 2014): the Caribbean, the inter-Andean valleys of the Cauca and Magdalena Rivers, the northern Andean Region in Santander including north Santander, the Patía River Valley and in rocky outcrops in the Departments of Arauca and Vichada in the Eastern Plains. This article focuses on the Colombian south-western tropical dry forest, which is located in the geographical valley of the Cauca River (VGRC), a region that has undergone extensive transformations, primarily due to the growth of the agricultural industry. In this area, sugar-cane cultivation has increased by 97% over the past decade (Uribe 2021) and livestock activities continue to dominate the VGRC landscape (Chacón de Ulloa et al. 2012). As a result, the VGRC landscape is characterised by forest remnants covering between 1.5% (Arcila Cardona et al. 2012) and 3% of its original cover (Torres et al. 2012), surrounded by extensive monocultures of sugar-cane and some pastures.

Ants play a fundamental role in ecosystem functioning due to their high specific, genetic, ecological and functional diversity. This diversity involves a broad range of ecological functions, including nutrient cycling, organic matter decomposition, seed dispersal, plant protection, pollination and pest control (Del Toro et al. 2015, Carvalho et al. 2020, Ng et al. 2021). Furthermore, ants are very abundant in most terrestrial ecosystems, both in terms of individuals and biomass (Schultheiss et al. 2022). They also occupy various trophic positions through complex interactions with other organisms (Helms et al. 2020). Their ubiquity and quick response to environmental stressors further reinforces their ecological importance (Parker and Kronauer 2021). Finally, ants are relatively easy to sample and monitor over short time periods (Agosti et al. 2000), a fact that makes them effective models for assessing environmental changes in natural ecosystems.

As noted above, ants are ecologically important to ecosystems through the diversity of functional services they can provide. However, ant communities and their ecosystem services can be affected by habitat loss resulting from landscape transformation and fragmentation (Arnan et al. 2018). Changes in vegetation structure influence food availability and alter competitive interactions, which can subsequently affect the characteristics and population dynamics of ant species (Hoffmann and Andersen 2003). In addition, not only are interactions between native species affected, but the arrival and establishment of non-native (introduced) ants can also occur, posing additional ecological challenges for local fauna. Changes in land use create favourable conditions for the establishment of these introduced species, which can generate significant impacts on local species (Blumenfeld et al. 2016). It is crucial to emphasise that the establishment and expansion of introduced species depend on their population growth, their ability to acclimatise to new environments and their competitive interactions with native species (Lach L. 2021). Consequently, the study of ants offers important insights into ecological complexity and the impacts of environmental disturbances, elucidating how these factors can transform ecosystem structure and dynamics.

Ant communities in the geographical valley of the Cauca River have been studied from various perspectives (Armbrecht and Chacón de Ulloa 1997, Ramírez et al. 2001, Achury et al. 2008, Chacón de Ulloa et al. 2012, Rivera Pedroza et al. 2019), generating information across spatial and temporal scales. The objective of this study is to assess the diversity and distribution of ant species across several TDF forest fragments and their adjacent productive matrices in the geographical valley of the Cauca River. The results provide valuable insights to to provide information for policy development for the protection, maintenance and conservation of this threatened ecosystem.

## Project description

### Title

Ant Assemblages: 27 Years of Variation in Fragments of the Cauca Valley Dry Forest and Surrounding Matrices, pertaining to the project "The Dry Forest in Context. Twenty-Five Years Later: Spatio-Temporal Relationships," within the programme "Multi-Scale Relationships in Biodiversity across Altitudinal Gradients in Tropical Forests (Minciencias-Universidad del Valle program code: 70306)".

### Personnel

Maria Alejandra Bautista-Giraldo and Inge Armbrecht.

### Funding

Francisco José de Caldas Autonomous Heritage National Fund for Financing Science, Technology and Innovation. Code: 1106-852-70306. Contact: No. 491-2020.

## Sampling methods

### Sampling description

Two sampling sessions were conducted at seven sites in the geographic valley of the Cauca River in Colombia. At each site, two types of habitats were sampled: a fragment of tropical dry forest (TDF) and its adjacent anthropogenic matrix. Of the seven sampled sites, three matrices consisted of industrialised sugar-cane crops, while four matrices corresponded to pastures (Table 1). At each site, four 90-metre transects were installed — two transects within the forest and two within the matrix. In the forest, transects were placed starting 20 m from the forest edge, whereas, in the matrix, transects were set 20 m from the matrix edge to control for edge effects. Within each habitat type, the two transects were separated by 70 m.

Each transect was divided into 10 equidistant stations and, at each station, six types of collection methods were employed as follows: (1) subterranean baiting, which involved placing bait inside a perforated Eppendorf tube buried 10 cm in the soil; (2) ground baiting, which consisted of a 20 × 10 cm piece of bond paper with bait on its surface, exposed on the ground; (3) arboreal baiting, similar to ground baiting, but with the bait folded within the paper and attached to a tree trunk or shrub (all baits consisted of approximately 1 cm³ of tuna in oil); (4) pitfall traps, consisting of a 170 ml cup buried flush with the ground and containing a solution with a 50:50 ratio of ethanol to water (both the baits and pitfall traps were left in place for 4.5 hours); (5) Mini-winkler, this consisted of collecting five litter samples covering each 1 m² around each station, i.e. five points on the periphery of the station were located, then the litter was placed in the sifting bag. The sifted litter was placed in a mini-winkler bag, leaving it to act for 48 hours; and (6) manual collection, which involved a six-minute search per station across all substrates.

Two sampling efforts were performed for each site, each occurring in the transition from dry season to rainy season. The first sampling phase took place between July and September 2021, while the second phase occurred between January and March 2022.

### Quality control

All collected ants were preserved in 96% alcohol, cleaned and identified to the level of species or morphospecies using an Olympus SZ61 stereoscope. For specimen identification, keys from Fernández et al. (2019) and the interactive LucidKey v.3.3 (Longino 2009), as well as online resources such as AntWeb (2024) and AntWiki (2024), were used. A portion of the specimens were pinned as a reference collection and deposited in the Museo de Entomología de la Universidad del Valle (MUSENUV). Given the large quantity of collected material, another portion was deposited at the Centro de Colecciones Biológicas de la Universidad de Magdalena (CBUMAG). All samples were collected under the general collection permit, Resolution 1070 of 28 August 2015, modified by Resolution 01255 of 28 June 2019, file IDB 0436-00.

## Geographic coverage

### Description

**Figure 1.**
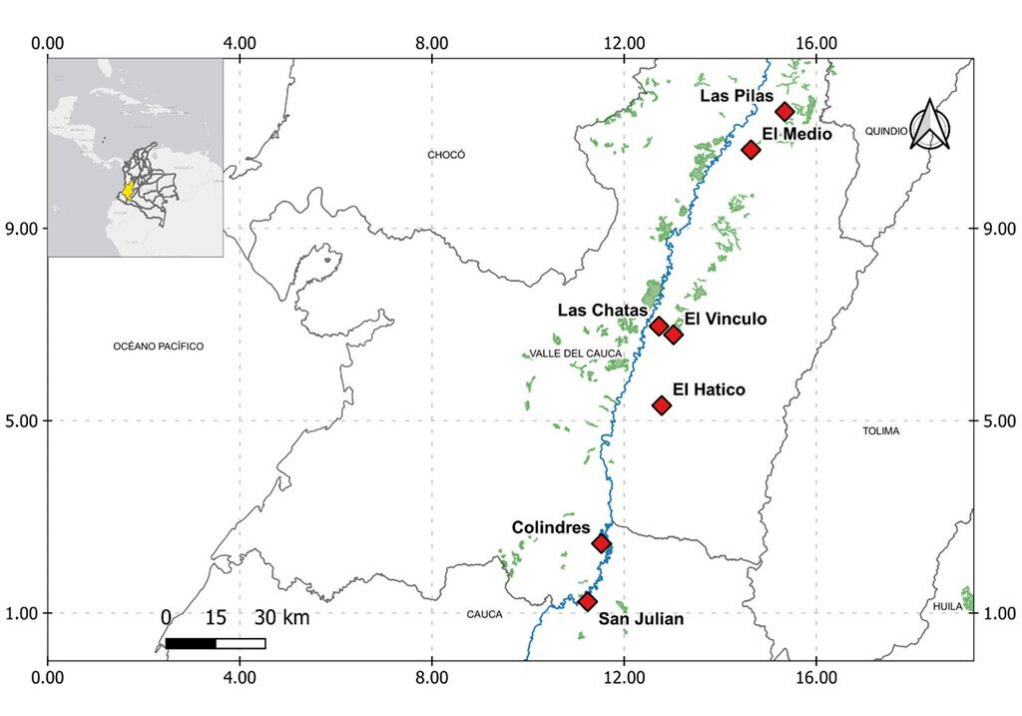
Location of the seven study sites in the geographic valley of the Cauca River. The blue line corresponds to the Cauca River and the green areas correspond to tropical dry forest (TDF). This map was created for this study.

The study was conducted at seven sites composed of forest fragments and adjacent anthropogenic matrices. These sites are located in south-western Colombia, throughout the geographic valley of the Cauca River (VGRC), between coordinates 3.10961 to 4.44062 and −76.52658 to −75.99042 (Fig. 1). The VGRC is an inter-Andean alluvial plain situated between the Central and Western mountain ranges (Reina Rodríguez et al. 2010). The valley is 230 km long and 10–20 km wide, with an elevation above sea level between 900 and 1200 m, an average temperature of 24°C and annual rainfall ranging from 1000 to 2000 mm (Arcila Cardona et al. 2012).

The area falls within the potential range of tropical dry forest (i.e. the site has the appropriate climatic conditions for TDF, but it does not necessarily retain its original vegetation), characterised by two rainy seasons (March–May and September–November) and two dry seasons (December–January and June–August) (Cuartas et al. 2017). It includes flood-prone areas and clayey soils favourable for agriculture, making it the main region in the Department for urban settlement and agro-industrial development (Urrutia 2005), particularly for sugar-cane cultivation and livestock farming activities.

## Taxonomic coverage

### Description

The dataset includes 25,022 individual ants across 2,120 records, distributed within two datasets: 24,476 individuals in 1574 records in the CBUMAG dataset and 546 individuals and records in the MUSENUV dataset (both are in accordance with the Darwin Core 4.0 format). In total, 229 species/morphospecies are reported, covering 54 genera and nine subfamilies. Of these, 124 species are shared across both databases, while 17 species are exclusive to the CBUMAG dataset and 88 to the MUSENUV dataset (Table 2). Of the 229 taxa, 81% were designated to species level and 19% to morphospecies level. Amongst the 186 identified species, 168 are native and nine are exotic species, which include *C.emeryi*, *C.wroughtonii*, *Monomoriumfloricola* (Jerdon, 1851), *Paratrechinalongicornis* (Latreille, 1802), *Pheidolemegacephala* (Fabricius, 1793), *S.emmae*, *Strumigenysrogeri* Emery, 1890, *Tapinomamelanocephalum* (Fabricius, 1793) and *Tetramoriumbicarinatum* (Nylander, 1846) (Table 2).

Some species, such as *Prionopeltaamabilis*/*antillana*, *Aztecatonduzi*/*chartifex* and *Solenopsistexana*/*zeteki*, could not be identified with a single specific epithet due to the lack of distinctive morphological characters in the available taxonomic keys. For other species, such as Apterostigmaaff.manni, Camponotusaff.cuneidorsus, Camponotusaff.pittieri, Brachymyrmexaff.degener, Hypoponeraaff.clavatula, Monomoriumaff.ebeninum, Pheidoleaff.angulifera, Pheidoleaff.diligens, Pheidoleaff.jamaicensis, Pheidoleaff.rugiceps, Pseudomyrmexaff.venustus, Rogeriaaff.curvipubens, Solenopsisaff.hayemi and Solenopsisaff.tenuis, taxonomic identities also remain unconfirmed, primarily due to morphological variations that do not match with established characters. Confirming the identities of these ants could represent new records for Colombia or potential range expansions of these species, which would contribute to more robust distribution models for Colombian ant fauna, enhance our understanding of ant biodiversity and significantly increase the recorded species richness in the country. Therefore, additional efforts are essential to clarify and confirm the taxonomic identity of these ants and expand available taxonomic resources.

In addition to previously reported exotic species in the Departments studied, we identified a range expansion of *Strumigenysemmae* (Emery, 1890) in the Department of Valle del Cauca, as well as *Cardiocondylawroughtonii* (Forel, 1890) and *Cardiocondylaemeryi* Forel, 1881 in the Department of Cauca. The record of *S.emmae* comes from the Colindres site, located in a municipality bordering northern Cauca, where this species had already been reported (Guerrero et al. 2018), suggesting a possible range expansion into Valle del Cauca. Similarly, *C.wroughtonii* and *C.emeryi*, previously reported in Valle del Cauca (Mackay 1995, Chacón de Ulloa et al. 2012), were found in Cauca, expanding their known distributions. Additionally, although *Pheidolemegacephala* had been previously reported in urban areas of Valle del Cauca (Fernández et al. 2019), we now report its presence in natural ecosystems, such as forest fragments, as well as in the surrounding anthropogenic matrices. Therefore, it is necessary to closely monitor the presence of this exotic species across different habitat types to understand the potential impact of that species on native biological diversity.

Overall, nine exotic species were recorded in the Cauca River Valley region (VGRC), indicating an increasing presence of non-native ants in this area. These findings highlight the importance of continued research on the distribution and population dynamics of exotic species, as well as the dissemination of such information, since many of these ants have high invasive potential (e.g. *Pheidolemegacephala*) (Lowe et al. 2004) and may pose significant threats to local biodiversity. Monitoring their dispersal and establishment patterns (i.e. potential naturalisation) is essential to prevent and mitigate possible ecological impacts.

## Usage licence

### Usage licence

Other

### IP rights notes

CC BY-NC 4.0

## Data resources

### Data package title

Ants from the tropical dry forest in the Cauca River Geographic Valley, Colombia: new records and expansion of distributions

### Resource link

https://www.gbif.org/dataset/d63054b1-2900-4230-9fb2-83a60b772d49 https://www.gbif.org/dataset/3cce7a63-aa6e-412c-a694-26947ab238c7

### Number of data sets

2

### Data set 1.

#### Data set name

Ants from Tropical Dry Forests and their surrounding matrices in the geographical valley of the Cauca River, which are deposited in the entomological collection of the Universidad del Magdalena.

#### Data format

Darwin Core Archive

#### Character set

Occurrence

#### Download URL

https://ipt.biodiversidad.co/sib/archive.do?r=umagdalena_hormigas-bosqueseco

#### Description

The dataset contains 1,574 records of ants and was published in the SiB Colombia as an open file under the Creative Commons Attribution 4.0 International licence. The terms used to name the fields follow the standard Darwin Core (Wieczorek et al. 2012, Darwin Core Maintenance Group 2023).

### Data set 2.

#### Data set name

Ants from Tropical Dry Forests and their surrounding matrices in the geographical valley of the Cauca River, which are deposited in the entomological collection of the Universidad de Valle.

#### Data format

Darwin Core Archive

#### Character set

Occurrence

#### Download URL

https://ipt.biodiversidad.co/sib/archive.do?r=univalle_hormigas_bosque-seco

#### Description

The dataset contains 546 records of ants and was published in the SiB Colombia as an open file under the Creative Commons Attribution 4.0 International licence. The terms used to name the fields follow the standard Darwin Core (Wieczorek et al. 2012, Darwin Core Maintenance Group 2023).

Below, following the Darwin Core version 4.0 record template, we list the columns and their descriptions used in both datasets. When downloading the files, two text documents will be available: "occurrence," which contains all Darwin Core columns, except those related to permits and "permit" which includes only the permit-related columns: *permitType*, *permitStatus* and *permitText*.

**Data set 2.** 

Column label	Column description
ocurrenceID	Unique identifier of the biological record.
basisOfRecord	Denotes the evidence from which the organism was derived. Only biological collections can document PreservedSpecimen.
type	Specifies the type of evidence that gave rise to the record.
institutionCode	Complete name of the institution that houses the specimens.
institutionID	Institution identifier registered in the institutionCode element.
collectionCode	Name or acronym that identifies the collection housing the records.
collectionID	Identifier of the collection that houses the records.
catalogNumber	Unique identifier assigned to the batch or specimen in the biological collection.
datasetName	Name of the dataset from which the biological records derive.
language	Language of the dataset.
recordNumber	Identifier of the biological record at the moment it was collected.
recordedBy	List of the names of the people, groups or organisations that collected the specimens.
recordedByID	List of the IDs of the people, groups or organisations registered in recordedBy.
individualCount	Number of individuals in the biological record.
sex	Sex of the organisms in the biological record.
lifeStage	Life stage of the organism when registered.
occurrenceStatus	Status indicating the presence or absence of specimens in the collection.
preparations	Conservation methods of the specimens.
disposition	Current status of the specimen in relation to the collection that houses the records.
samplingProtocol	Sampling protocol of the biological record.
eventDate	Date or date range when the specimens were collected or the sampling event took place. This includes additional columns, such as year, month and day.
habitat	Habitat or description of the habitat where the specimens were collected.
continent	Name of the continent where the specimens were collected.
country	Name of the country where the specimens were collected.
countryCode	Standard code for the country of the location.
stateProvince	Full name of the next administrative region of lower hierarchy in the country where the specimens were collected (Department).
county	Full name of the next administrative region of lower hierarchy after Department.
locality	More specific geographic information on where the specimens were collected.
minimumElevationInMetres	Lower limit of the elevation range where the specimens were collected.
maximumElevationInMetres	Upper limit of the elevation range where the specimens were collected.
verbatimLatitude	Original latitude of the location where the specimens were collected.
verbatimLongitude	Original longitude of the location where the specimens were collected.
verbatimCoordinates	Coordinates of the location where the specimens were collected in their original format.
establishmentMeans	Statement about whether a dwc:Organism has been introduced to a given place and time through the direct or indirect activity of modern humans.
decimalLatitude	Latitude in decimal degrees.
decimalLongitude	Longitude in decimal degrees.
geodeticDatum	The ellipsoid, geodetic datum or spatial reference system (SRS) on which the geographic coordinates provided in decimalLatitude and decimalLongitude are based.
georeferencedBy	List of individuals, groups or organisations that determined the georeference for the location where specimens were collected.
georeferenceSources	List of maps, gazetteers or other resources used to georeference the collection location of the specimens.
georeferenceRemarks	Comments or notes regarding the spatial description.
identifiedBy	List of individuals who identified the specimen(s).
identifiedByID	Identifiers (ORCID or Wikidata) of the people who identified the organism.
identificationRemarks	Comments or notes regarding the identification.
verbatimIdentification	Original identification of the specimen.
identificationQualifier	Degree of uncertainty in the identification, such as aff. (means affine) to the scientific name.
scientificName	Canonical scientific name corresponding to the taxonomic category to which the specimen was identified.
scientificNameAuthorship	Authorship information corresponding to scientificName.
kingdom	Kingdom to which the specimen belongs.
phylum	Phylum to which the taxon belongs.
class	Class to which the taxon belongs.
order	Order to which the taxon belongs.
family	Family to which the taxon belongs.
subfamily	Subfamily to which the taxon belongs.
genus	Genus to which the taxon belongs.
specificEpithe	Specific epithet of the taxon.
taxonRank	Taxonomic category of the name present in scientificName.
vernacularName	Common name of the taxon.
coordinateUncertaintyInMetres	The horizontal distance (in metres) from the given dwc:decimalLatitude and dwc:decimalLongitude describing the smallest circle containing the whole of the dcterms:Location.
eventID	An identifier for the set of information associated with a dwc:Event (something that occurs at a place and time). May be a global unique identifier or an identifier specific to the dataset.
infraspecificEpithet	The name of the lowest or terminal infraspecific epithet of the dwc:scientificName, excluding any rank designation.
taxonomicStatus	The status of the use of the dwc:scientificName as a label for a taxon.
permitTyp (Permit extension)	A permit is a document that allows someone to take an action that otherwise would not be allowed.
permitStatus (Permit extention)	Information about the presence, absence or other basic status of permits associated with the sample(s).
permitText (Permit extension)	The text of a permit related to the gathering/shipping or further details.

## Figures and Tables

**Table 1.** Description, altitude and coordinates of the sampled sites in the geographic valley of the Cauca River. The sites are ordered from south to north. Industrialised sugar-cane is defined as large-scale agricultural fields that use heavy machinery and chemical inputs. Treed pastures contained trees more than three decades old, approximately 60 trees/hectare. Open pasture contained sporadic trees or lacked tree coverage. Regenerating pastures contained approximately 60 trees/hectare from one to six metres. **Note**: *The geographic coordinates of Hacienda Las Pilas seem to correspond to the County of La Victoria; however, this site has historically been documented as pertaining to the County of Zarzal, Valle del Cauca.

**Site**	**County**	**Department**	**Elevation (m)**	**Latitude**	**Longitude**	**Anthropogenic Matrix**
Hacienda Nanaluisa San Julián	Santander de Quilichao	Cauca	954	3.10961	−76.52658	Industrialised sugar-cane
Reserva de la Sociedad Civil Bosque Colindres	Jamundí	Valle del Cauca	970	3.26714	−76.48661	Industrialised sugar-cane
Reserva Natural El Hatico	El Cerrito	Valle del Cauca	988	3.64189	−76.32537	Treed pasture
Parque Natural Regional El Vínculo	Guadalajara de Buga	Valle del Cauca	983	3.83600	−76.29830	Treed pasture
Bosque Seco Tropical Inundable De Las Chatas (humedal continental)	Guadalajara de Buga	Valle del Cauca	943	3.85681	−76.33067	Regenerating pasture
Hacienda El Medio	Zarzal	Valle del Cauca	943	4.33481	−76.08081	Industrialised sugar-cane
Hacienda Las Pilas*	Zarzal	Valle del Cauca	991	4.44062	−75.99042	Open pasture

**Table 2.** Species and morphospecies correspond to each of the registered ant genera and subfamilies. Species in bold and labelled with ** indicate new records for Colombia. The collections where the specimens are deposited are identified with the following acronyms: CBUMAG (Centro de Colecciones Biológicas at the Universidad del Magdalena), MUSENUV (Museo de Entomología at the Universidad del Valle) and CBUMAG/MUSENUV for those deposited in both collections. Biogeographic status is classified as native, exotic or uncertain; in the case of morphospecies, the status of the morphologically similar species was considered. In those cases where a morphospecies could not be defined, the taxonomic identity of the genus was considered. The Departments where the specimens are found are indicated as follows: VC = Valle del Cauca, C = Cauca and * indicates a distribution expansion for the Departments.

**Subfamily**	**Genus**	**Species or morphospecies**	**Collection**	**Biogeographic status**	**Distribution in the TDF sampled**
Agroecomyrmecinae	*Tatuidris*	*Tatuidristatusia* Brown & Kempf, 1968	CBUMAG/MUSENUV	Native	VC
Amblyoponinae	*Prionopelta*	*Prionopeltaamabilis*/*antillana*	CBUMAG/MUSENUV	Native	VC
		*Prionopelta* sp. 1	MUSENUV	Native	C
Dolichoderinae	*Azteca*	*Aztecaalfari* Emery, 1893	CBUMAG/MUSENUV	Native	VC*
		*Aztecavelox* Forel, 1899	CBUMAG/MUSENUV	Native	VC, C*
		*Aztecatonduzi*/*chartifex*	CBUMAG/MUSENUV	Native	VC
	*Dolichoderus*	*Dolichoderusbispinosus* (Olivier, 1792)	CBUMAG/MUSENUV	Native	VC
		*Dolichoderusdiversus* Emery, 1894	MUSENUV	Native	VC
		*Dolichoderuslutosus* (Smith, 1858)	CBUMAG	Native	VC
	*Dorymyrmex*	*Dorymyrmexbrunneus* Forel, 1908	MUSENUV	Native	VC
	*Forelius*	*Foreliusdamiani* Guerrero & Fernández, 2008	CBUMAG/MUSENUV	Native	VC*
	*Linepithema*	*Linepithemainiquum* (Mayr, 1870)	CBUMAG/MUSENUV	Native	VC, C
		*Linepithemaneotropicum* Wild, 2007	CBUMAG	Native	VC
	*Tapinoma*	*Tapinomamelanocephalum* (Fabricius, 1793)	MUSENUV	Exotic	C
		*Tapinomaramulorum* Emery, 1896	MUSENUV	Native	VC
Dorylinae	*Eciton*	*Ecitonmexicanum* Roger, 1863	MUSENUV	Native	VC*
	*Labidus*	*Labiduscoecus* (Latreille, 1802)	CBUMAG/MUSENUV	Native	VC
		*Labidusspininodis* (Emery, 1890)	CBUMAG/MUSENUV	Native	VC
	*Neivamyrmex*	*Neivamyrmexemersoni* (Wheeler, 1921)	MUSENUV	Native	VC*
	*Nomamyrmex*	*Nomamyrmexhartigii* (Westwood, 1842)	MUSENUV	Native	VC
Ectatomminae	*Ectatomma*	*Ectatommaruidum* (Roger, 1860)	CBUMAG/MUSENUV	Native	VC
	*Gnamptogenys*	*Gnamptogenysannulata* (Mayr, 1887)	CBUMAG/MUSENUV	Native	VC
		*Gnamptogenyshorni* (Santschi, 1929)	CBUMAG	Native	VC
	*Holcoponera*	*Holcoponeramoelleri* Forel, 1912	CBUMAG/MUSENUV	Native	VC*
Formicinae	*Acropyga*	*Acropygaexsanguis* (Wheeler, 1909)	CBUMAG/MUSENUV	Native	VC, C
		*Acropygafuhrmanni* (Forel, 1914)	CBUMAG/MUSENUV	Native	VC
	*Brachymyrmex*	*Brachymyrmexaustralis* Forel, 1901	CBUMAG/MUSENUV	Native	VC
		*Brachymyrmexcordemoyi* Forel, 1895	CBUMAG/MUSENUV	Native	VC*, C*
		*Brachymyrmexminutus* Forel, 1893	CBUMAG/MUSENUV	Native	VC, C*
		*Brachymyrmexmusculus* Forel, 1899	CBUMAG/MUSENUV	Native	VC*
		*Brachymyrmexobscurior* Forel, 1893	CBUMAG/MUSENUV	Native	VC
		Brachymyrmexaff.degener	MUSENUV	Native	VC
	*Camponotus*	*Camponotusatriceps* (Smith, 1858)	CBUMAG/MUSENUV	Native	VC
		*Camponotusbidens* Mayr, 1870	CBUMAG/MUSENUV	Native	VC
		*Camponotusbrevis* Forel, 1899	CBUMAG/MUSENUV	Native	VC
		*Camponotusexcisus* Mayr, 1870	CBUMAG/MUSENUV	Native	VC
		*Camponotusindianus* Forel, 1879	CBUMAG/MUSENUV	Native	VC, C
		*Camponotuslindigi* Mayr, 1870	CBUMAG/MUSENUV	Native	VC, C
		*Camponotuslinnaei* Forel, 1886	MUSENUV	Native	VC
		*Camponotusnovogranadensis* Mayr, 1870	CBUMAG/MUSENUV	Native	VC
		*Camponotussenex* (Smith, 1858)	CBUMAG/MUSENUV	Native	VC, C
		Camponotusaff.cuneidorsus	MUSENUV	Native	VC
		Camponotusaff.pittieri	MUSENUV	Native	VC
		*Camponotus* sp. 1	MUSENUV	Native	VC, C
		*Camponotus* sp. 2	MUSENUV	Native	VC
		*Camponotus* sp. 3	MUSENUV	Native	VC
		*Camponotus* sp. 4	MUSENUV	Native	VC
		*Camponotus* sp. 5	MUSENUV	Native	VC
		*Camponotus* sp. 6	MUSENUV	Native	C
		*Camponotus* sp. 7	MUSENUV	Native	VC
		*Camponotus* sp. 8	MUSENUV	Native	VC
	*Nylanderia*	*Nylanderiaguatemalensis* (Forel, 1885)	CBUMAG/MUSENUV	Native	VC
		*Nylanderiasteinheili* (Forel, 1893)	CBUMAG	Native	VC
		*Nylanderia* sp. 1	MUSENUV	Native	VC
	*Paratrechina*	*Paratrechinalongicornis* (Latreille, 1802)	CBUMAG/MUSENUV	Exotic	VC, C
Myrmicinae	*Acanthognathus*	*Acanthognathusbrevicornis* Smith, 1944	CBUMAG/MUSENUV	Native	VC
		*Acanthognathusocellatus* Mayr, 1887	CBUMAG/MUSENUV	Native	VC, C
		*Acanthognathusteledectus* Brown & Kempf, 1969	MUSENUV	Native	VC
	*Apterostigma*	*Apterostigmacarinatum* Lattke, 1997	CBUMAG/MUSENUV	Native	VC
		*Apterostigmapilosum*	CBUMAG/MUSENUV	Native	VC
		Apterostigmaaff.manni	MUSENUV	Native	VC
	*Atta*	*Attacephalotes* (Linnaeus, 1758)	CBUMAG/MUSENUV	Native	VC
	*Cardiocondyla*	*Cardiocondylaemeryi* Forel, 1881	CBUMAG/MUSENUV	Exotic	VC, C*
		*Cardiocondylanuda* (Mayr, 1866)	CBUMAG/MUSENUV	Uncertain	VC
		*Cardiocondylawroughtonii* (Forel, 1890)	CBUMAG/MUSENUV	Exotic	VC, C*
	*Carebara*	*Carebarabrevipilosa* Fernández, 2004	CBUMAG/MUSENUV	Native	VC
	*Cephalotes*	*Cephalotesbasalis* (Smith, 1876)	CBUMAG/MUSENUV	Native	VC
		*Cephalotescristatus* (Emery, 1890)	MUSENUV	Native	VC
		*Cephalotesmaculatus* (Smith, 1876)	CBUMAG/MUSENUV	Native	VC
		*Cephalotesminutus* (Fabricius, 1804)	CBUMAG/MUSENUV	Native	VC
		*Cephalotesporrasi* (Wheeler, 1942)	MUSENUV	Native	VC*
		*Cephalotestargionii* (Emery, 1894)	MUSENUV	Native	VC*
		*Cephalotesumbraculatus* (Fabricius, 1804)	MUSENUV	Native	VC*
	*Crematogaster*	*Crematogasterabstinens* Forel, 1899	CBUMAG/MUSENUV	Native	VC
		*Crematogasterampla* Forel, 1912	CBUMAG/MUSENUV	Native	VC
		*Crematogasterbrasiliensis* Mayr, 1878	CBUMAG/MUSENUV	Native	VC
		*Crematogastercarinata* Mayr, 1862	CBUMAG/MUSENUV	Native	VC
		*Crematogastercrinosa* Mayr, 1862	CBUMAG/MUSENUV	Native	VC
		*Crematogastercurvispinosa* Mayr, 1862	CBUMAG/MUSENUV	Native	VC, C
		*Crematogasterdistans* Mayr, 1870	MUSENUV	Native	VC
		*Crematogastererecta* Mayr, 1866	MUSENUV	Native	VC
		*Crematogasterevallans* Forel, 1907	CBUMAG/MUSENUV	Native	VC, C*
		*Crematogastermontezumia* Smith, 1858	CBUMAG/MUSENUV	Native	VC*
		*Crematogasternigropilosa* Mayr, 1870	CBUMAG/MUSENUV	Native	VC
		*Crematogasterobscurata* Emery, 1895	MUSENUV	Native	VC*
		*Crematogastersotobosque* Longino, 2003	CBUMAG/MUSENUV	Native	VC
		*Crematogastertenuicula* Forel, 1904	CBUMAG/MUSENUV	Native	VC
		*Crematogastertorosa* Mayr, 1870	CBUMAG/MUSENUV	Native	VC
	*Cyphomyrmex*	*Cyphomyrmexcostatus* Mann, 1922	CBUMAG/MUSENUV	Native	VC
		*Cyphomyrmexminutus* Mayr, 1862	CBUMAG/MUSENUV	Native	VC, C
		*Cyphomyrmexrimosus* (Spinola, 1851)	CBUMAG/MUSENUV	Native	VC, C
	*Hylomyrma*	*Hylomyrma* sp. 1	MUSENUV	Native	VC
	*Megalomyrmex*	*Megalomyrmexdrifti* Kempf, 1961	CBUMAG/MUSENUV	Native	VC, C*
		*Megalomyrmex* sp. 1	MUSENUV	Native	VC
		*Megalomyrmex* sp. 2	MUSENUV	Native	VC
		*Megalomyrmex* sp. 3	MUSENUV	Native	VC
	*Monomorium*	*Monomoriumfloricola* (Jerdon, 1851)	CBUMAG/MUSENUV	Exotic	VC, C
		Monomoriumaff.ebeninum	MUSENUV	Uncertain	VC
	*Mycetomoellerius*	*Mycetomoelleriusopulentus* (Mann, 1922)	CBUMAG/MUSENUV	Native	VC
	*Mycocepurus*	*Mycocepurussmithii* (Forel, 1893)	CBUMAG	Native	VC
	*Nesomyrmex*	*Nesomyrmexasper* (Mayr, 1887)	CBUMAG/MUSENUV	Native	VC
		*Nesomyrmexechinatinodis* (Forel, 1886)	CBUMAG/MUSENUV	Native	VC, C
		*Nesomyrmexpittieri* (Forel, 1899)	MUSENUV	Native	VC*
	*Octostruma*	*Octostrumabalzani* (Emery, 1894)	CBUMAG/MUSENUV	Native	VC
		*Octostrumabatesi* (Emery, 1894)	CBUMAG/MUSENUV	Native	VC*
		*Octostrumaexcertirugis* Longino, 2013	MUSENUV	Native	VC*
		*Octostrumaobtusidens* Longino, 2013	MUSENUV	Native	VC*
		*Octostruma* sp. 3	MUSENUV	Native	VC
		*Octostruma* sp. 4	MUSENUV	Native	VC
		*Octostruma* sp. 5	MUSENUV	Native	VC
	*Paratrachymyrmex*	*Paratrachymyrmexcornetzi* (Forel, 1912)	CBUMAG/MUSENUV	Native	VC, C
	*Pheidole*	*Pheidolebiconstricta* Mayr, 1870	CBUMAG/MUSENUV	Native	VC
		*Pheidolebilimeki* Mayr, 1870	CBUMAG/MUSENUV	Native	VC*
		*Pheidoleboliviana* Wilson, 2003	CBUMAG/MUSENUV	Native	VC*
		*Pheidolecataractae* Wheeler, 1916	CBUMAG/MUSENUV	Native	VC
		*Pheidolecolobopsis* Mann, 1916	MUSENUV	Native	VC*
		*Pheidolefimbriata* Roger, 1863	CBUMAG/MUSENUV	Native	VC, C*
		*Pheidoleflavens* Roger, 1863	CBUMAG/MUSENUV	Native	VC, C
		*Pheidolegauthieri* Forel, 1901	CBUMAG/MUSENUV	Native	VC*
		*Pheidolehasticeps* Wilson, 2003	MUSENUV	Native	VC*
		*Pheidolelongiscapa* Forel, 1901	CBUMAG/MUSENUV	Native	VC, C*
		*Pheidolemegacephala* (Fabricius, 1793)	CBUMAG/MUSENUV	Exotic	VC
		*Pheidoleradoszkowskii* Mayr, 1884	CBUMAG/MUSENUV	Native	VC, C*
		*Pheidolesculptior* Forel, 1893	CBUMAG/MUSENUV	Native	VC*, C*
		*Pheidoleseeldrayersi* Forel, 1910	MUSENUV	Native	VC
		*Pheidolesimonsi* Wilson, 2003	CBUMAG/MUSENUV	Native	VC*
		*Pheidolesubarmata* Mayr, 1884	CBUMAG/MUSENUV	Native	VC*
		*Pheidolesusannae* Forel, 1886	CBUMAG/MUSENUV	Native	VC
		*Pheidolesynarmata* Wilson, 2003	CBUMAG/MUSENUV	Native	VC
		Pheidoletraini** Wilson, 2003	MUSENUV	Uncertain	VC
		*Pheidoletransversostriata* Mayr, 1887	CBUMAG	Native	VC
		*Pheidolevallifica* Forel, 1901	CBUMAG/MUSENUV	Native	VC*
		Pheidoleaff.angulifera	CBUMAG/MUSENUV	Native	VC
		Pheidoleaff.diligens	MUSENUV	Native	VC
		Pheidoleaff.jamaicensis	MUSENUV	Uncertain	C
		Pheidoleaff.rugiceps	CBUMAG/MUSENUV	Native	VC, C
		*Pheidole* sp. 11	MUSENUV	Native	C
		*Pheidole* sp. 20	MUSENUV	Native	VC
		*Pheidole* sp. 21	MUSENUV	Native	VC
		*Pheidole* sp. 27	MUSENUV	Native	VC
		*Pheidole* sp. 33	MUSENUV	Native	VC
		*Pheidole* sp. 34	MUSENUV	Native	VC
	*Procryptocerus*	*Procryptocerusferreri* Forel, 1912	MUSENUV	Native	VC
		*Procryptocerusscabriusculus* Forel, 1899	CBUMAG/MUSENUV	Native	VC
	*Rogeria*	*Rogeriaforeli* Emery, 1894	CBUMAG/MUSENUV	Native	VC, C
		*Rogeriagibba* Kugler, 1994	CBUMAG/MUSENUV	Native	VC
		*Rogeriascandens* (Mann, 1922)	CBUMAG/MUSENUV	Native	VC, C*
		Rogeriaaff.curvipubens	MUSENUV	Native	VC
	*Solenopsis*	*Solenopsisazteca* Forel, 1893	CBUMAG/MUSENUV	Native	VC*, C
		*Solenopsisbicolor* (Emery, 1906)	CBUMAG/MUSENUV	Native	VC, C
		*Solenopsisbrevicornis* Emery, 1888	CBUMAG/MUSENUV	Native	VC*, C
		*Solenopsisgeminata* (Fabricius, 1804)	CBUMAG	Native	VC
		*Solenopsispicea* Emery, 1896	CBUMAG/MUSENUV	Native	VC
		*Solenopsisrugiceps* Mayr, 1870	MUSENUV	Native	VC
		*Solenopsisstricta* Emery, 1896	CBUMAG/MUSENUV	Native	VC
		*Solenopsisvinsoni* Pacheco & Mackay, 2013	CBUMAG/MUSENUV	Native	VC
		Solenopsisaff.hayemi	MUSENUV	Native	VC
		Solenopsisaff.tenuis	MUSENUV	Native	VC, C
		*Solenopsistexana*/*zeteki*	CBUMAG/MUSENUV	Native	VC, C
		*Solenopsis* sp. 9	MUSENUV	Native	VC
	*Strumigenys*	*Strumigenysbiolleyi* Forel, 1908	CBUMAG/MUSENUV	Native	VC, C
		*Strumigenysdenticulata* Mayr, 1887	CBUMAG	Native	VC
		*Strumigenyseggersi* Emery, 1890	CBUMAG/MUSENUV	Native	VC
		*Strumigenysemmae* (Emery, 1890)	MUSENUV	Exotic	VC*
		*Strumigenysgrytava* (Bolton, 2000)	MUSENUV	Native	VC*
		*Strumigenysgundlachi* (Roger, 1862)	CBUMAG/MUSENUV	Native	VC, C
		*Strumigenyslouisianae* Roger, 1863	CBUMAG	Native	VC
		*Strumigenysmarginiventris* Santschi, 1931	CBUMAG	Native	VC*
		*Strumigenysperparva* Brown, 1958	CBUMAG/MUSENUV	Native	VC
		*Strumigenysrogeri* Emery, 1890	CBUMAG	Exotic	C
		*Strumigenyssmithii* Forel, 1886	MUSENUV	Native	VC
		*Strumigenyssubedentata* Mayr, 1887	CBUMAG/MUSENUV	Native	VC
		*Strumigenystrinidadensis* Wheeler, 1922	CBUMAG/MUSENUV	Native	VC, C
		*Strumigenyswheeleriana* Baroni Urbani, 2007	MUSENUV	Native	VC*
		*Strumigenyszeteki* (Brown, 1959)	CBUMAG/MUSENUV	Native	VC
	*Temnothorax*	*Temnothoraxsubditivus* (Wheeler, 1903)	MUSENUV	Native	VC*
	*Tetramorium*	*Tetramoriumbicarinatum* (Nylander, 1846)	CBUMAG/MUSENUV	Exotic	VC, C
	*Tranopelta*	*Tranopeltagilva* Mayr, 1866	CBUMAG/MUSENUV	Native	VC
	*Wasmannia*	*Wasmanniaauropunctata* (Roger, 1863)	CBUMAG	Native	VC, C
		Wasmanniasulcaticeps** Emery, 1894	MUSENUV	Uncertain	VC
Ponerinae	*Anochetus*	*Anochetusdiegensis* Forel, 1912	MUSENUV	Native	VC
		*Anochetusmayri* Emery, 1884	CBUMAG/MUSENUV	Native	VC, C
	*Hypoponera*	*Hypoponeradistinguenda* (Emery, 1890)	CBUMAG	Native	VC
		*Hypoponerafiebrigiantoniensis* (Forel, 1912)	CBUMAG/MUSENUV	Native	VC*, C*
		*Hypoponerafoeda* (Forel, 1893)	MUSENUV	Native	VC
		*Hypoponeraopaciceps* (Mayr, 1887)	CBUMAG/MUSENUV	Native	VC
		*Hypoponeraopacior* (Forel, 1893)	CBUMAG/MUSENUV	Native	VC*
		*Hypoponeraparva* (Forel, 1909)	CBUMAG/MUSENUV	Native	VC*, C*
		*Hypoponeratrigona* (Mayr, 1887)	CBUMAG/MUSENUV	Native	VC
		Hypoponeraaffclavatula	MUSENUV	Native	VC
	*Leptogenys*	Leptogenysmontuosa** Lattke, 2011	MUSENUV	Uncertain	VC
		*Leptogenyspubiceps* Emery, 1890	MUSENUV	Native	VC*
	*Mayaponera*	*Mayaponeraconstricta* (Mayr, 1884)	CBUMAG/MUSENUV	Native	VC
		*Mayaponera* sp. 1	MUSENUV	Native	VC
	*Neoponera*	*Neoponeraapicalis* (Latreille, 1802)	MUSENUV	Native	VC
		*Neoponeracarinulata* (Roger, 1861)	CBUMAG/MUSENUV	Native	VC
		*Neoponeracrenata* (Roger, 1861)	CBUMAG/MUSENUV	Native	VC
		*Neoponerafoetida* (Linnaeus, 1758)	MUSENUV	Native	VC
		*Neoponeramoesta* Mayr, 1870	MUSENUV	Native	VC
		Neoponerarugosula** Emery, 1902	CBUMAG/MUSENUV	Uncertain	VC, C
		*Neoponeraunidentata* Mayr, 1862	MUSENUV	Native	VC
		Neoponerazuparkoi** Mackay & Mackay, 2010	CBUMAG	Uncertain	VC
		*Neoponeraverenae* (Forel, 1922)	CBUMAG/MUSENUV	Native	VC, C
	*Odontomachus*	*Odontomachuschelifer* (Latreille, 1802)	CBUMAG/MUSENUV	Native	VC
		*Odontomachuserythrocephalus* Emery, 1890	CBUMAG/MUSENUV	Native	VC
		*Odontomachushaematodus* (Linnaeus, 1758)	MUSENUV	Native	C
		*Odontomachusruginodis* Smith, 1937	CBUMAG	Native	VC
	*Pachycondyla*	*Pachycondylaharpax* (Fabricius, 1804)	CBUMAG/MUSENUV	Native	VC
		*Pachycondylaimpressa* (Roger, 1861)	CBUMAG	Native	VC
	*Thaumatomyrmex*	*Thaumatomyrmexatrox* Weber, 1939	MUSENUV	Native	VC
Pseudomyrmecinae	*Pseudomyrmex*	*Pseudomyrmexboopis* (Roger, 1863)	MUSENUV	Native	VC
		*Pseudomyrmexelongatus* (Mayr, 1870)	CBUMAG/MUSENUV	Native	VC
		*Pseudomyrmexfiliformis* (Fabricius, 1804)	CBUMAG/MUSENUV	Native	VC
		*Pseudomyrmexgebellii* (Forel, 1899)	MUSENUV	Native	VC
		*Pseudomyrmexgracilis* (Fabricius, 1804)	CBUMAG/MUSENUV	Native	VC
		*Pseudomyrmexita* (Forel, 1906)	MUSENUV	Native	VC
		*Pseudomyrmexkuenckeli* (Emery, 1890)	CBUMAG/MUSENUV	Native	VC
		*Pseudomyrmexlongior* (Forel, 1904)	MUSENUV	Native	VC*
		*Pseudomyrmexlongus* (Forel, 1912)	MUSENUV	Native	VC
		*Pseudomyrmexoculatus* (Smith, 1855)	CBUMAG	Native	VC
		*Pseudomyrmexoki* (Forel, 1906)	MUSENUV	Native	VC
		*Pseudomyrmexpallens* (Mayr, 1870)	MUSENUV	Native	VC
		*Pseudomyrmexsimplex* (Smith, 1877)	CBUMAG/MUSENUV	Native	VC
		*Pseudomyrmextenuissimus* (Emery, 1906)	CBUMAG/MUSENUV	Native	VC
		*Pseudomyrmextermitarius* (Smith, 1855)	CBUMAG/MUSENUV	Native	VC, C
		*Pseudomyrmex* psw005	MUSENUV	Native	VC
		*Pseudomyrmex* psw015	MUSENUV	Native	VC
		*Pseudomyrmex* aff. psw005	MUSENUV	Native	VC
		Pseudomyrmexaff.venustus	MUSENUV	Native	VC
TOTAL		229			
